# Cumambrin B Alleviates Sepsis-Associated Acute Lung Injury by Activating the Nrf2/HO-1 Pathway

**DOI:** 10.3390/biomedicines14030729

**Published:** 2026-03-23

**Authors:** Yuemei Que, Dandan Ruan, Minxia Xu, Ying Nie, Guozheng Huang, Huajun Zhao, Yanzi Yang

**Affiliations:** 1 School of Pharmaceutical Sciences, Zhejiang Chinese Medical University, Hangzhou 311402, China; 2 School of Chemistry and Chemical Engineering, Anhui University of Technology, Ma’anshan 243002, China; 3 Academy of Chinese Medical Sciences, Zhejiang Chinese Medical University, Hangzhou 310053, China

**Keywords:** sepsis-associated acute lung injury, Cumambrin B, inflammation, oxidative stress, mitochondrial function, Nrf2/HO-1 pathway

## Abstract

**Background:** Sepsis-associated acute lung injury (SA-ALI) is a prevalent complication observed in patients with severe infection, characterized primarily by uncontrolled inflammatory response. Cumambrin B (CB) is a natural sesquiterpene lactone with anti-inflammatory properties. However, its protective effects against SA-ALI and the underlying molecular mechanisms remain unclear. **Methods:** Mice that received intraperitoneal lipopolysaccharide (LPS) injection were used to assess the protective effect of CB on SA-ALI. LPS-induced RAW264.7 cells were utilized to delve into the molecular mechanisms responsible for its protective effects. **Results:** CB markedly alleviated lung tissue injury in mice with SA-ALI. Network pharmacology and combined in vitro and in vivo studies demonstrated that the protective effect of CB against SA-ALI was closely related to its anti-inflammatory and antioxidant activities. Meanwhile, CB restored mitochondrial function and activated the nuclear factor erythroid 2-related factor 2/heme oxygenase-1 (Nrf2/HO-1) signaling pathway. Furthermore, Nrf2 activator potentiated the inhibitory effects of CB on inflammation and oxidative stress, whereas these effects were abolished by an Nrf2 inhibitor. **Conclusions:** CB alleviates SA-ALI by restoring mitochondrial function, attenuating oxidative stress and inflammation via activation of the Nrf2/HO-1 pathway.

## 1. Introduction

Sepsis is a life-threatening multi-organ dysfunction syndrome caused by the body’s abnormal response to infection, and it is one of the leading causes of death among patients in intensive care units [1,2,3]. Globally, the incidence of sepsis is high, with over 30 million reported cases each year and an overall mortality rate of 20–30% [1,4]. However, effective intervention strategies are still lacking in clinical practice. In the multi-organ damage caused by sepsis, the lungs are the earliest and most susceptible target organ [5,6]. Thus, those affected by sepsis are prone to develop acute lung injury (ALI), which can progress to acute respiratory distress syndrome (ARDS) in severe cases [7]. Clinically, sepsis-associated acute lung injury (SA-ALI) primarily manifests as refractory hypoxemia, with its core features being the damage to the alveolar–capillary barrier, accompanied by an extensive infiltration of inflammatory cells, the release of inflammatory mediators, and pulmonary edema [8,9,10]. SA-ALI and its progressive form, ARDS, are core contributing factors to mortality in patients with sepsis [11]. Clinical data show that over 50% of sepsis patients will develop ALI, and once it progresses to ARDS, the mortality rate within 90 days can soar to 35.5% [12,13,14]. However, there are currently no definitive treatments available, highlighting the urgent need to identify effective therapeutic agents to alleviate SA-ALI.

While a moderate inflammatory response serves as an essential defense mechanism for pathogen clearance and tissue repair, excessive or uncontrolled inflammation can lead to significant lung tissue damage [15]. In SA-ALI, inflammatory cells such as neutrophils infiltrate the lungs in large numbers, concomitant with excessive production of pro-inflammatory cytokines. Consequently, the balance between pro-inflammatory and anti-inflammatory responses is disrupted, resulting in the amplification of the inflammatory cascade which ultimately exacerbates lung damage [16]. Oxidative stress, stemming from the excessive accumulation of reactive oxygen species (ROS) predominantly generated by mitochondria, is another key contributor to the pathogenesis of SA-ALI [17,18]. Physiological levels of ROS play a crucial role in microbial defense; however, elevated ROS levels during sepsis can directly damage mitochondrial DNA, proteins, and lipids, impairing mitochondrial structural integrity and functional stability, which induce mitochondrial dysfunction and exacerbate lung injury [9]. Notably, inflammation and oxidative stress are closely interconnected: inflammatory cytokines promote ROS generation, whereas oxidative stress upregulates pro-inflammatory gene expression. This bidirectional interaction weaves a “positive feedback web” that perpetuates tissue injury and disease in a cycle of continuous damage. Accordingly, targeting both inflammation and oxidative stress, or disrupting their interaction, suggests a feasible therapeutic option for SA-ALI.

Cumambrin B (CB) was confirmed in early studies as a sesquiterpene lactone, initially identified in 1968 from *Ambrosia cumanensis* HBK [19]. Additionally, CB has been shown to be extractable from the flowers of *Chrysanthemum boreale* [20]. Our previous research found that CB could be successfully isolated and purified from *Ajania fruticulosa* (Ledeb.) Poljak and showed nitric oxide (NO) inhibitory activity [21]. *Ajania fruticulosa* is a plant species within the genus *Ajania* of the Asteraceae family, recognized for its diverse pharmacological functions, including anti-inflammatory and antioxidant properties [22]. Our prior studies revealed that CB blocks inflammatory responses in lipopolysaccharide (LPS)-stimulated macrophages and ALI caused by LPS intratracheal instillation through suppressing the nuclear factor-kappa B (NF-κB) and mitogen-activated protein kinase (MAPK) signaling pathways [23]. Therefore, building on our preliminary findings, the present study further investigates the effects of CB on lung injury induced by systemic inflammation secondary to sepsis, as well as the underlying in-depth molecular mechanisms.

The nuclear factor erythroid 2-related factor 2 (Nrf2) signaling pathway is recognized as a central defensive mechanism against oxidative stress and mitochondrial damage. Under basal conditions, Nrf2 is sequestered in the cytoplasm and subjected to proteasomal degradation. Upon exposure to oxidative stress, Nrf2 translocates into the nucleus, where it binds to antioxidant response elements (ARE) and transcriptionally activates a series of cytoprotective genes, including heme oxygenase-1 (HO-1) and NAD(P)H:quinone oxidoreductase 1 (NQO1) [24,25]. In addition, Nrf2 signaling activation is capable of lowering ROS levels, thereby preserving the steady state of mitochondrial function [26,27]. Given that CB exhibited favorable anti-inflammatory and antioxidant activities, the present study further explored the association between its role in SA-ALI and its ability to restore mitochondrial function and activate the Nrf2/HO-1 signaling pathway.

Our research aimed to explore CB’s role in attenuating SA-ALI, centering on the anti-inflammatory, antioxidant, and mitochondrial function-restoring effects. We found that CB exerts these effects via activating the Nrf2/HO-1 signaling pathway. Our study identifies CB’s protective function and mechanistic pathways in SA-ALI, pointing to its potential therapeutic value for relieving pulmonary injury.

## 2. Materials and Methods

### 2.1. Animals

The study employed 36 male C57BL/6 mice (aged between 6 and 8 weeks) as experimental subjects. These mice were obtained from Zhejiang Vital River Laboratory Animal Technology Co., Ltd. (Jiaxing, China) (Specific Pathogen Free [SPF] grade, Certification No.: 20241016Abzz0600080859). Animal housing services were provided by the Experimental Animal Research Center of Zhejiang Chinese Medical University (Laboratory Animal Use License No.: SYXK (Zhe) 2019-0024). The Institutional Animal Care and Use Committee conducted a review and gave approval to the experimental protocol (Approval No.: PHA-IACUC-20241021-01, Date: 5 August 2025).

After 3 days of acclimatization housing under SPF conditions, the 36 mice underwent random allocation into 6 groups, respectively the control group, the LPS group, the LPS + CB (5 mg/kg) group, the LPS + CB (10 mg/kg) group, the LPS + CB (20 mg/kg) group, and the LPS + Dexamethasone (Dex, 5 mg/kg) group. Over a period of seven consecutive days, each mouse received intraperitoneal injections of its respective treatment (CB, Dex, or an equivalent volume of saline) on a daily basis. On the 7th day, one hour post-administration, SA-ALI was triggered via intraperitoneal LPS injection (5 mg/kg), whereas the vehicle group was administered the identical quantity of saline. After the LPS injection 18–24 h, the mice were euthanized, bronchoalveolar lavage fluid (BALF) and lung tissues were collected for subsequent experiments.

### 2.2. Reagents and Chemicals

CB (purity ≥ 98%) was kindly provided by the research group of Professor Guo-Zheng Huang at Anhui University of Technology. Dex (cat. no. A601187-0005) was sourced from Sangon Biotech Co., Ltd. (Shanghai, China), and LPS (cat. no. L2880) was obtained from Sigma-Aldrich (St. Louis, MO, USA). All remaining materials and reagents can be found in Supplementary Table S1.

### 2.3. Method of Drug Dissolution

CB was dissolved in dimethyl sulfoxide (DMSO) (cat. no. V900090, Sigma-Aldrich, St. Louis, MO, USA) to prepare a stock solution and then diluted with physiological saline for mouse administration or diluted with the culture medium for cell experiments. The final concentration of DMSO in the culture medium was controlled below 0.1% to ensure no cytotoxicity occurred.

### 2.4. Network Pharmacology

First, confirm the chemical structure of CB on PubChem (accessed on 29 December 2025: https://pubchem.ncbi.nlm.nih.gov/), then use SwissTargetPrediction (accessed on 29 December 2025: http://www.swisstargetprediction.ch/) to screen potential drug targets containing CB. Using “acute lung injury” as the keyword, search GeneCards 5.23 (accessed on 29 December 2025: https://genealacart.genecards.org/), OMIM (accessed on 29 December 2025: https://www.omim.org/), Digenet (accessed on 29 December 2025: https://disgenet.com/) and TTD (accessed on 29 December 2025: http://db.idrblab.net/ttd/) to screen disease-related genes. Next, we use Venny 2.1 (accessed on 29 December 2025: https://bioinfogp.cnb.csic.es/tools/venny/) to remove duplicates and perform intersection analysis between these genes and drug targets to determine potential targets for CB in regulating ALI. These intersection targets are then imported into Metascape (accessed on 29 December 2025: https://metascape.org/) for pathway enrichment analysis, and the resulting data are visualized using Wei Sheng Xin (accessed on 29 December 2025: https://www.bioinformatics.com.cn/).

### 2.5. Cell Culture

The murine macrophage cell line RAW264.7 was obtained from the Cell Bank of the Chinese Academy of Sciences (Shanghai, China). Cells were grown in a high-glucose Dulbecco’s modified Eagle medium (DMEM) supplemented with 10% fetal bovine serum and maintained in a 5% CO_2_ environment at 37 °C.

### 2.6. Thiazolyl Blue Tetrazolium Bromide (MTT) Assay

Based on existing reports, the cytotoxic effect of CB on RAW264.7 cells was analyzed using the MTT method [23]. A density of 1 × 10^4^ cells per well was used to seed 96-well plates, which were then treated with CB at multiple concentrations over 24 h. Subsequently, 20 μL of MTT solution was added to each well and incubated for 4 h. Upon completion of the incubation, 100 μL of crystal dissolution solution was introduced into every well. Measurements of absorbance were taken at 570 nm with a microplate reader (BioTek, Winooski, VT, USA).

### 2.7. Real-Time Quantitative Polymerase Chain Reaction (RT-qPCR)

For the experiment, a seeding density of 6 × 10^5^ cells per well was used for RAW264.7 cells in 6-well plates, followed by 24 h of incubation. First, the cells were exposed to CB (2, 4 and 8 μM) for 3 h; then, LPS stimulation (1 μg/mL) was applied for 24 h. Total RNA was isolated from harvested cells using the total RNA extraction kit (cat. no. K156001, Thermo Fisher Scientific, Waltham, MA, USA) and then reverse-transcribed into cDNA using the reverse transcription kit (cat. no. AG11706, Accurate Biotechnology, Changsha, China). Subsequently, RT-qPCR was performed on a real-time PCR instrument (Applied Biosystems, Foster City, CA, USA). First, a 5 min denaturation at 95 °C was performed. Next, 40 cycles were carried out, including denaturation (95 °C, 20 s), annealing (55 °C, 20 s), and extension (72 °C, 15 s). Finally, a 5 min extension at 72 °C completed the procedure. We primarily used RT-qPCR to detect the expression of inflammatory cytokines interleukin-6 (*IL-6*), interleukin-1β (*IL-1β*), tumor necrosis factor-α (*TNF-α*), and interleukin-18 (*IL-18*) in lung tissues and RAW264.7 cells. The primer sequences involved in this experiment were shown in Table 1.

### 2.8. Analysis Using Assay Kits

Following the same pretreatment procedure as described for RT-qPCR, the cells were harvested. The malondialdehyde (MDA) and glutathione (GSH) levels, along with superoxide dismutase (SOD) activity, were then measured according to the manufacturers’ instructions (cat. no. A003-1-2, A006-2-1, and A001-3-2, respectively; Nanjing Jiancheng Bioengineering Institute, Nanjing, China). Meanwhile, MDA, GSH levels, and SOD activity in lung tissues were quantified using the kit’s recommended procedures.

### 2.9. Flow Cytometric Analysis

The experiment involved plating 6 × 10^5^ RAW264.7 cells per well in 6-well plates to measure intracellular ROS levels. The cells underwent 3 h of CB pretreatment with 2, 4 and 8 μM before being added with 1 μg/mL LPS for 24 h. Post-treatment, the cells were washed using phosphate-buffered saline (PBS) and then they were labeled for 30 min in the dark with 500 μL of PBS containing 50 μM DCFH-DA (cat. no. S1105S, Beyotime Biotechnology, Shanghai, China). Following three PBS washes and centrifugation to clear the DCFH-DA probe, flow cytometry was employed to assess fluorescence intensity. Both mitochondrial ROS (cat. no. S0061S, Beyotime Biotechnology, Shanghai, China) accumulation and mitochondrial membrane potential (cat. no. C2005, Shanghai, China) were analyzed via flow cytometry (Beckman Coulter, Brea, CA, USA).

### 2.10. Oxygen Consumption Rate (OCR) Analysis

Excessive accumulation of ROS can impair mitochondrial function. To investigate whether LPS stimulation affects mitochondrial function, the harvested cells were subjected to PBS washes before being resuspended in DMEM culture medium, and counted. Cellular OCR was then measured using an OCR assay kit (cat. no. 023004, Beijing Huawei Zhongyi Technology Co., Ltd., Beijing, China) and the oroboros O2k high-resolution respirometry system (Oroboros Instruments GmbH, Innsbruck, Austria).

### 2.11. Immunofluorescence Assay

After exposure to CB and LPS as described above, the confocal dishes containing 6 × 10^4^ cells were fixed with 4% paraformaldehyde for 15 min, 0.5% Triton X-100 for permeabilization for 15 min, and were used with 1% bovine serum albumin (BSA) to block them at room temperature for 1 h. The dishes underwent two incubation steps: the sample was treated with the primary Nrf2 antibody overnight at 4 °C, and then for 2 h in the dark with a fluorescent secondary antibody. Then, the nuclei were counterstained with 4′,6-diamidino-2-phenylindole (DAPI) (cat. no. D9542, Sigma-Aldrich, St. Louis, MO, USA) for 10 min. Finally, the slides were immediately observed and imaged under a laser confocal microscope (Beckman Coulter, Brea, CA, USA).

### 2.12. Enzyme-Linked Immunosorbent Assay (ELISA)

Collect the supernatant from RAW264.7 cells that have been pretreated with CB and then stimulated with LPS. Use a mouse *IL-1β* ELISA kit (cat. no. MM-0040M2, Wuhan Enzyme-Linked Biotechnology Co., Ltd., Wuhan, China) to detect the release of *IL-1β*.

### 2.13. Western Blot

RAW264.7 cells were cultured in 6-well plates at 6 × 10^5^ cells per well and treated with CB at 2, 4, and 8 μM for 3 h, followed by stimulation with LPS (1 μg/mL) for 24 h. Cells were subsequently harvested, lysed with the radio-immunoprecipitation assay lysis buffer, and then homogenized. Following centrifugation of the homogenate at 16,000× *g* for 15 min, the supernatant was harvested for protein quantification. Cell proteins were obtained by boiling with 1× loading buffer, separated by 10% SDS-polyacrylamide gel electrophoresis, and then transferred onto polyvinylidene fluoride (PVDF) membranes. The membranes were incubated with primary antibodies overnight at 4 °C. After retrieval of the primary antibodies and washing, the membranes were incubated with secondary antibody diluted 1:3000 for 2 h, followed by detection using enhanced chemiluminescence substrate. Protein expression levels of Nrf2 (cat. no. ab62352, 1:1000, Abcam, Cambridge, MA, USA), NQO1 (cat. no. ab80588, 1:1000, Abcam, Cambridge, MA, USA), and HO-1 (cat. no. 43966S, 1:1000, Cell Signaling Technology, Danvers, MA, USA) on the membranes were analyzed with GAPDH (cat. no. 60004-1, 1:1000, Proteintech, Rosemont, IL, USA) used as an internal loading control.

### 2.14. Nuclear and Cytoplasmic Fractionation

Cells were processed as described in the Western blot section. Nuclear and cytoplasmic proteins were separated using the nuclear and cytoplasmic protein extraction kit (cat. no. P0028, Beyotime Biotechnology, Shanghai, China) according to the manufacturer’s instructions. Subsequently, the expression levels of Nrf2 protein in the nuclear and cytoplasmic fractions were determined using Western blot, with GAPDH as a cytoplasmic internal reference and histone H3 (H3) (cat. no. ab309551, 1:1000, Abcam, Cambridge, MA, USA) as a nuclear protein internal reference.

### 2.15. Measurement of Lung Wet-to-Dry Weight Ratio (W/D)

Following euthanasia, the left lung was surgically excised from each mouse, briefly rinsed with PBS, and blotted dry on filter paper to remove surface moisture. An electronic analytical balance was employed to accurately measure and record the fresh weight of lung tissues. After drying the tissue at 65 °C for 72 h in an oven, the dry weight was assessed, and the W/D was derived.

### 2.16. Collect BALF from Mice

After euthanasia, the thoracic and cervical cavities were opened to expose the trachea. Following tracheal cannulation, the fluid was then aspirated using a syringe attached to the catheter, and this lavage operation was carried out on three occasions. Centrifugation of the collected BALF was performed at 1500× *g* for 10 min to separate cells from the supernatant. Total protein concentration in the samples was quantified via a bicinchoninic acid (BCA) protein assay kit (cat. no. P0011, Beyotime Biotechnology, Shanghai, China). The cell pellet was gently resuspended in 100 μL of PBS, and the total cells, including leukocytes and neutrophils, were counted using a hemocytometer.

### 2.17. Hematoxylin and Eosin (H&E) Staining

After fixation, the murine lung samples were dehydrated, placed into paraffin blocks, and sliced into sections. H&E staining was carried out using a dedicated kit (cat. no. C0105, Beyotime Biotechnology, Shanghai, China), followed by mounting with neutral balsam. Once dried, the lung tissues underwent observation of pathological changes and photography via a Zeiss upright fluorescence microscope (Zeiss, Oberkochen, Germany).

### 2.18. Statistical Analysis

Using Excel 2019 and GraphPad Prism 8.0.2, data were analyzed statistically and expressed as mean ± SD ($\bar{X}$ ± SD). Mean group differences were assessed with Student’s *t*-test, with *p* < 0.05 considered significant.

## 3. Results

### 3.1. CB Attenuates SA-ALI in Mice

**Figure 1:**
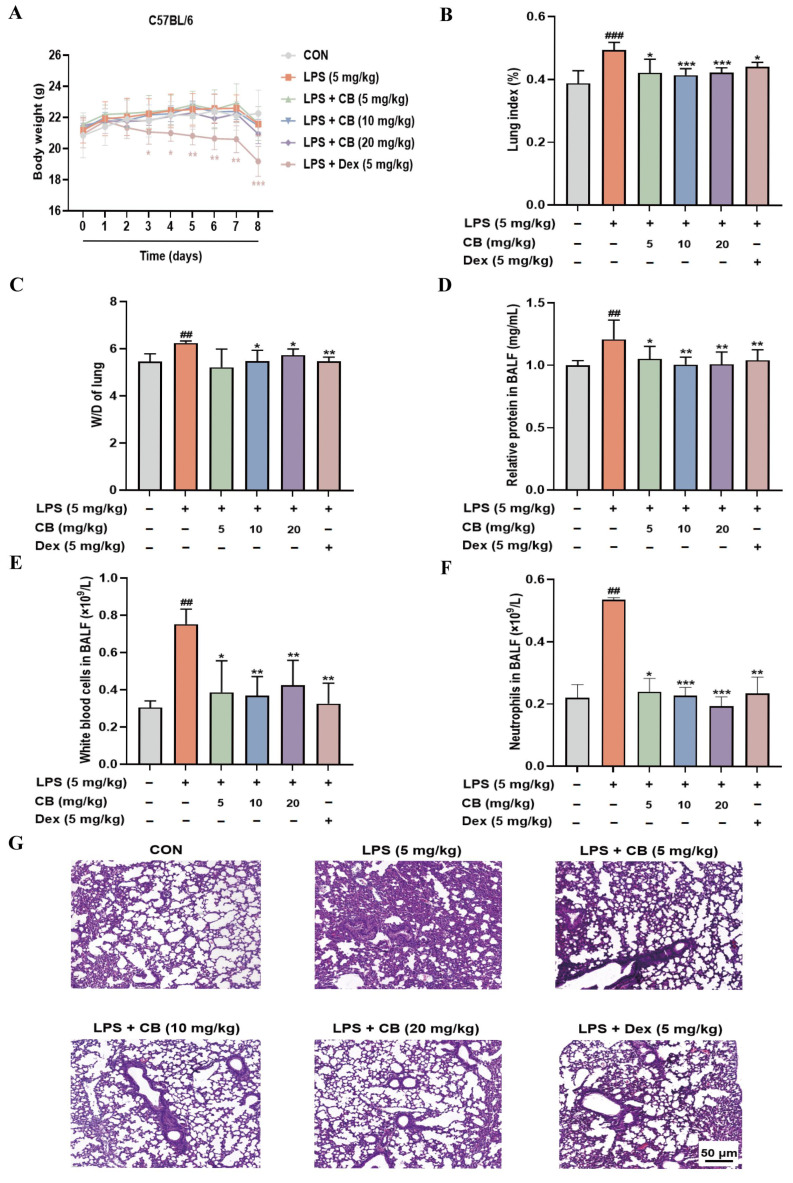
CB alleviates SA-ALI in mice. (**A**) Body weights of mice during administration; (**B**) Lung index measurements; (**C**) The lung W/D; (**D**) Total protein in BALF; (**E**) White blood cells in BALF; (**F**) Neutrophils in BALF; (**G**) H&E staining images. The magnification used for the H&E staining images was 20×, with the scale bars representing 50 µm. BALF: Bronchoalveolar lavage fluid; CB: Cumambrin B; CON: Control group; Dex: Dexamethasone; LPS: Lipopolysaccharide; W/D: Wet-to-dry weight ratio. (* versus LPS, # versus CON, * *p* < 0.05; ** *p* < 0.01; *** *p* < 0.001; ## *p* < 0.01; ### *p* < 0.001).

We conducted an initial evaluation of CB’s protective role against lung using an SA-ALI model. No remarkable variations in body weight were found between the CB-treated and the normal mice throughout the experiment, except for the Dex-treated ones (Figure 1A). Pulmonary edema severity is reflected through the lung tissue W/D and BALF total protein content. As expected, the LPS challenge significantly increased pulmonary vascular permeability, with evidence from raised W/D and enhanced protein leakage in BALF. In contrast, CB pretreatment markedly attenuated these LPS-induced changes (Figure 1B–D). Furthermore, since neutrophil infiltration and leukocyte retention can exacerbate inflammatory responses and cellular damage, we detected inflammatory cells in BALF using a blood cell counter. A notable reduction in total leukocytes and neutrophils in the BALF was observed following CB pretreatment (Figure 1E,F). H&E staining revealed clear pulmonary tissue structure, intact alveolar architecture, and no significant pathological changes in the control group. In contrast, the LPS group exhibited partial alveolar collapse and significant thickening of the alveolar walls (Figure 1G). Collectively, these data imply that CB acts protectively against SA-ALI in mice.

### 3.2. CB Inhibits Inflammatory Response in LPS-Induced SA-ALI Mice Lung Tissue and RAW264.7 Cells

**Figure 2:**
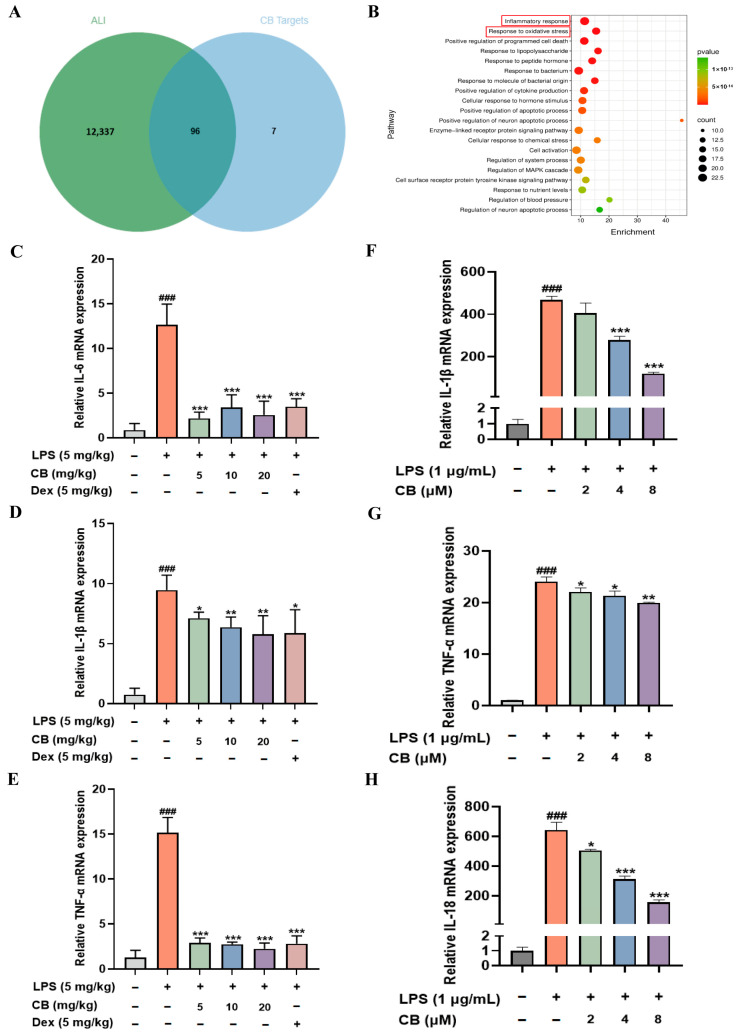
CB suppresses the expression of inflammatory cytokines induced by LPS. (**A**) Venn diagram of overlapping targets between CB-related targets and ALI targets; (**B**) GO enrichment bubble chart, the red box used to highlight the key enriched pathways; (**C**–**E**) The mRNA levels of *IL-6*, *IL-1β*, and *TNF-α* in mouse lung tissue from different treatment groups, as determined by RT-qPCR; (**F**–**H**) The mRNA levels of *IL-1β*, *TNF-α* and *IL-18* in RAW264.7 cells were quantified using RT-qPCR. ALI: Acute lung injury; CB: Cumambrin B; Dex: Dexamethasone; LPS: Lipopolysaccharide. (* versus LPS, # versus CON, * *p* < 0.05; ** *p* < 0.01; *** *p* < 0.001; ### *p* < 0.001).

Network pharmacology studies have confirmed that inflammation and oxidative stress are the key factors through which CB protects the organism from ALI-induced damage. Based on the screening of 12,433 disease-related genes and 103 drug targets, network pharmacology analysis identified 96 potential target genes associated with ALI (Figure 2A). GO enrichment analysis revealed that inflammatory response and oxidative stress were the top-ranked pathways (Figure 2B), indicating their critical roles in mediating CB’s protection against ALI caused by LPS-induced sepsis. The essence of SA-ALI is the concentrated manifestation and severe consequence of a systemic inflammatory response in the lungs. Consequently, controlling the excessive inflammatory response is one of the fundamental therapeutic strategies for this condition. CB’s anti-inflammatory activity was investigated through cytokine expression measurement in murine lung tissue, revealing significant suppression of *IL-6*, *IL-1β*, and *TNF-α* (Figure 2C–E). Additional studies in RAW264.7 cells assessed cytokine levels after LPS exposure, with findings consistent with the in vivo model. (Figure 2F–H). Thus, these findings demonstrated notable anti-inflammatory activity of CB.

### 3.3. CB Alleviates Oxidative Stress in SA-ALI and LPS-Induced RAW264.7 Cells

**Figure 3:**
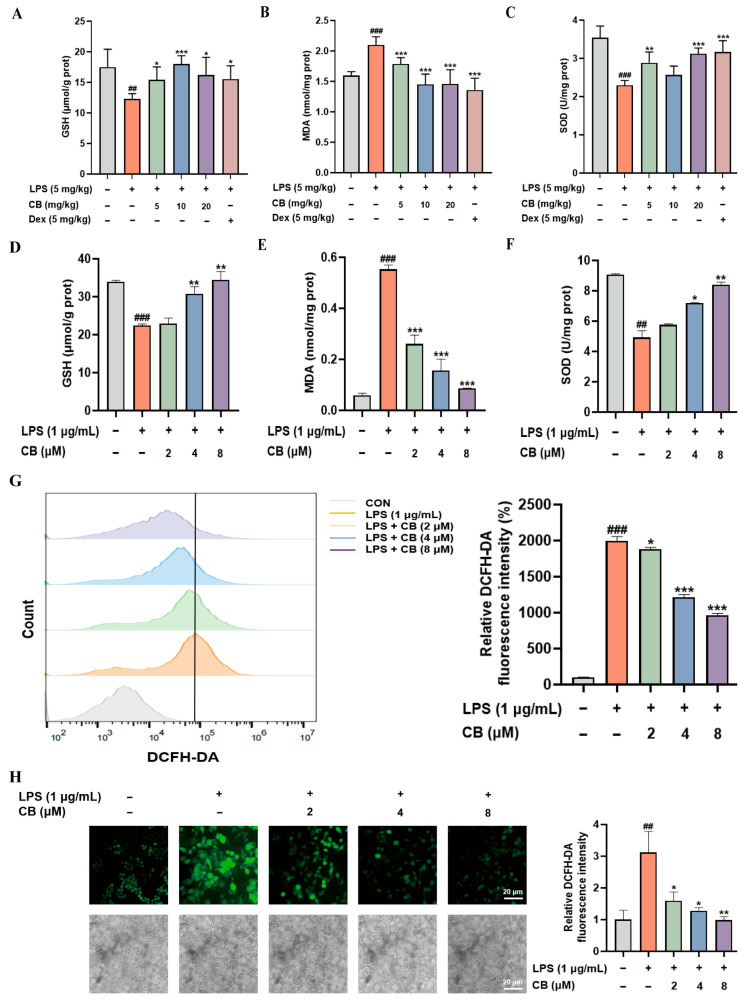
CB inhibits oxidative stress in SA-ALI and LPS-induced RAW264.7 cells. (**A**–**C**) Measurement of GSH and MDA levels, SOD activity in mouse lung tissue using commercial assay kits; (**D**–**F**) Measurement of GSH and MDA levels, SOD activity in RAW264.7 cells using commercial assay kits; (**G**) Flow cytometry analysis of the effect of CB on LPS-induced ROS production in RAW264.7 cells; (**H**) Fluorescence microscopy analysis of the effect of CB on LPS-induced ROS production in RAW264.7 cells. The magnification used for the images was 40×, with the scale bars representing 20 µm. CB: Cumambrin B; CON: Control group; Dex: Dexamethasone; GSH: Glutathione; LPS: Lipopolysaccharide; MDA: Malondialdehyde; SOD: Superoxide dismutase. (* versus LPS, # versus CON, * *p* < 0.05; ** *p* < 0.01; *** *p* < 0.001; ## *p* < 0.01; ### *p* < 0.001).

Within the pathogenic mechanisms of ALI caused by sepsis, the organism produces ROS through various pathways at levels far exceeding the physiological range, leading to the onset of oxidative stress. However, excessive oxidative stress is detrimental to normal lung physiology. This prompted us to investigate whether CB possesses antioxidant activity. Assessment of oxidative stress biomarkers like MDA, GSH, and SOD in lung tissue revealed that LPS administration reduced GSH levels and SOD activity but increased MDA levels. Conversely, CB treatment effectively reversed these alterations (Figure 3A–C). LPS stimulation led to consistent results in RAW264.7 cell assays (Figure 3D–F). ROS serves as a commonly used metric in the assessment of oxidative stress. So, we investigate intracellular ROS levels by DCFH-DA staining. As expected, LPS treatment markedly increased ROS levels, while CB pretreatment significantly reduced them (Figure 3G). This result was further confirmed by fluorescence microscopy (Figure 3H). Collectively, these results indicate the pronounced antioxidant activity of CB.

### 3.4. CB Attenuates Mitochondrial Dysfunction in LPS-Induced RAW264.7 Cells

**Figure 4:**
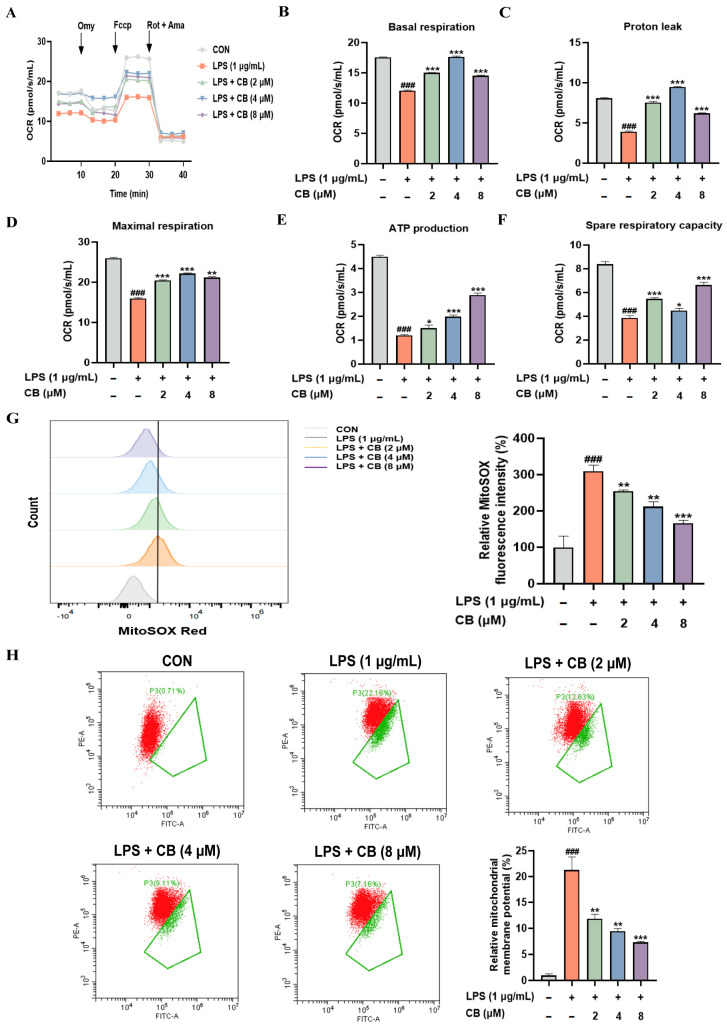
CB ameliorates mitochondrial dysfunction caused by LPS. (**A**) Mitochondrial OCR in RAW264.7 cells was measured by the O2k system; (**B**) Basal respiration; (**C**) Proton leak; (**D**) Maximal respiration; (**E**) ATP production; (**F**) Spare respiratory capacity; (**G**,**H**) Flow cytometric analysis of mitochondrial ROS levels and mitochondrial membrane potential in LPS-induced RAW264.7 cells treated with CB. CB: Cumambrin B; CON: Control group; LPS: Lipopolysaccharide; OCR: Oxygen consumption rate. (* versus LPS, # versus CON, * *p* < 0.05; ** *p* < 0.01; *** *p* < 0.001; ### *p* < 0.001).

Research indicates that mitochondrial dysfunction is a crucial factor in the progression of SA-ALI disease [28,29,30]. Both the primary generation of oxidative stress and its most critical target are attributed to mitochondria. Excessive production of ROS induces oxidative stress, which damages mitochondrial components and leads to further deterioration of mitochondrial function. This mitochondrial dysfunction, in turn, triggers an explosive increase in ROS production, thereby creating a vicious cycle [31]. The measurement of cellular oxygen consumption rate, a direct readout of mitochondrial electron transport chain activity and cellular energy status, is the “gold standard” for assessing mitochondrial function. Therefore, we measured cellular oxygen consumption using a cell energy metabolism analysis system (Figure 4A). LPS stimulation impaired basal respiration (Figure 4B), proton leak (Figure 4C), maximal respiration (Figure 4D), ATP production (Figure 4E) and spare respiratory capacity (Figure 4F). However, CB intervention improved these impairments. Furthermore, mitochondrial ROS activity and membrane potential results corroborated oxygen consumption data (Figure 4G,H). LPS-triggered mitochondrial impairment in RAW264.7 cells is ameliorated by CB, as evidenced by the above outcomes.

### 3.5. CB Alleviates SA-ALI via the Activation of the Nrf2/HO-1 Signaling

**Figure 5:**
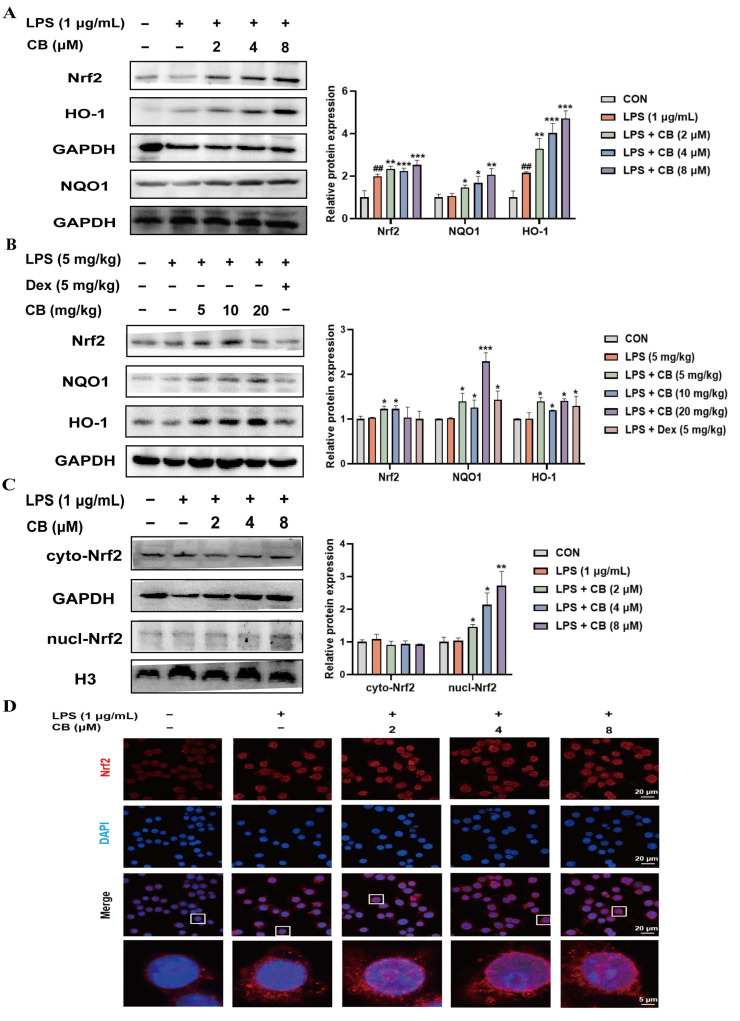
CB alleviates SA-ALI by activating the Nrf2/HO-1 signaling pathway. (**A**) The expression of Nrf2 and its associated proteins in RAW264.7 cells was analyzed by Western blot; (**B**) Western blot was used to detect the expression of Nrf2-related proteins in lung tissues; (**C**) The expression of Nrf2 protein in nuclear protein was detected by Western blot; (**D**) Observation of Nrf2 nuclear translocation by immunofluorescence. The white box in the figure represents the area where the cell nucleus below was enlarged. The oil immersion magnification used for the images was 63×, with the scale bars representing 20 µm, and the scale for the enlarged cell nucleus is 5 µm. CB: Cumambrin B; CON: Control group; DAPI: 4′,6-diamidino-2-phenylindole; Dex: Dexamethasone; LPS: Lipopolysaccharide. (* versus LPS, # versus CON, * *p* < 0.05; ** *p* < 0.01; *** *p* < 0.001; ## *p* < 0.01).

Nrf2, functioning as a multifunctional regulator, curtails inflammatory responses and decreases oxidative harm, thus critically modulating the pathways involved in ALI pathogenesis [32,33]. The Nrf2 signaling cascade, when activated, improves cellular antioxidant protection, curbs inflammatory processes, and lessens tissue harm [34]. HO-1 is one of the most important and representative target genes under Nrf2 regulation. Following Nrf2 activation, the transcription and expression of HO-1 are significantly upregulated [35]. Based on the antioxidant properties of CB, we further investigated its effect on the Nrf2 pathway. Western blot results revealed that CB promotes Nrf2, HO-1, and NQO1 expression (Figure 5A,B). The nuclear protein results showed that CB promotes Nrf2 nuclear translocation, which was also consistently observed by fluorescence microscope (Figure 5C,D). These results indicate that CB confers protection against ALI associated with sepsis through activation of the Nrf2/HO-1 pathway.

**Figure 6:**
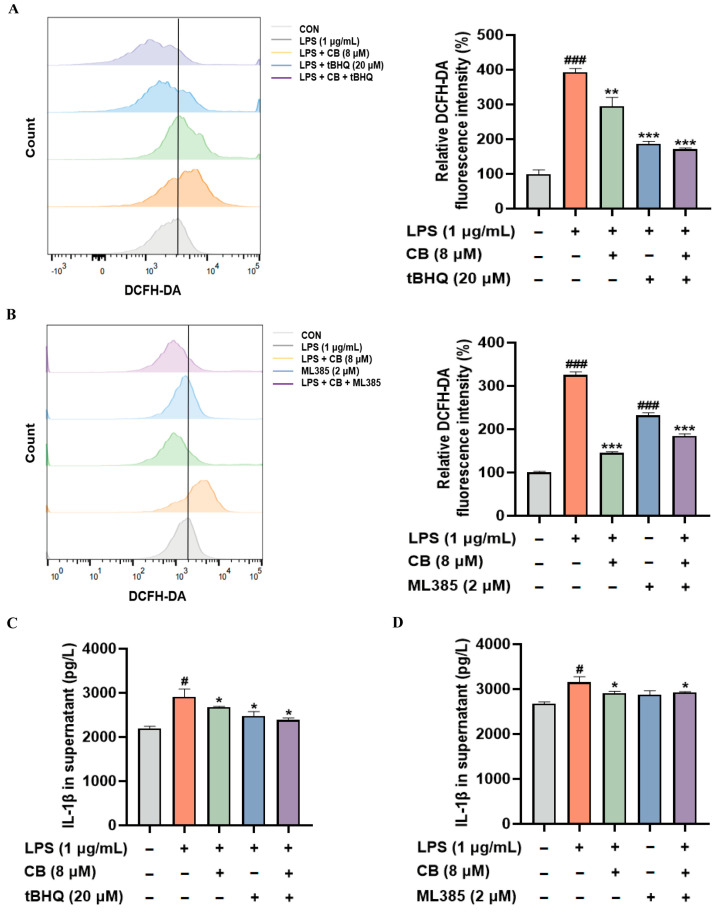
CB exerts a protective effect through the activation of the Nrf2 signaling pathway. (**A**,**B**) Intracellular ROS levels were measured by flow cytometry using DCFH-DA fluorescence; (**C**,**D**) The IL-1β level in the cell culture supernatant was determined by ELISA. CB: Cumambrin B; CON: Control group; LPS: Lipopolysaccharide. (* versus LPS, # versus CON, * *p* < 0.05; ** *p* < 0.01; *** *p* < 0.001; # *p* < 0.05; ### *p* < 0.001).

For the purpose of clarifying the contribution of Nrf2 to CB’s protective actions against inflammatory and oxidative stress responses, either ML385 (an Nrf2 inhibitor) or tertiary butylhydroquinone (tBHQ) (an Nrf2 activator) was used to treat RAW264.7 cells. We found that CB suppressed LPS-induced ROS production and IL-1β release in RAW264.7 cells. These suppressive effects were reversed by the Nrf2 inhibitor ML385 and enhanced by the Nrf2 activator tBHQ (Figure 6A–D). These research findings confirm that the Nrf2 signaling pathway activation serves as the main pathway for CB to achieve its beneficial function.

## 4. Discussion

SA-ALI is a life-threatening condition characterized by a high prevalence and significant fatal outcomes [36]. The essence of sepsis lies in the dysregulation of the body’s immune response triggered by an infection. Following a local infection, pathogens invade the bloodstream, leading to overactivation of the immune system. This is followed by the onset of a “cytokine storm”, ultimately culminating in damage to organs including the lungs. SA-ALI is significantly influenced by excessive inflammatory activity, which acts as a key driver of disease progression [32,37]. Macrophage hyperactivation during inflammation plays a significant role in contributing to immune dysfunction in SA-ALI [17]. Our research found that CB improved the degree of pulmonary edema, the protein leakage in BALF, the infiltration of white blood cells and neutrophils, and reduced lung tissue pathological damage. At the same time, CB can also inhibit the expression of inflammatory factors *IL-6*, *IL-1β*, and *TNF-α*, alleviating the inflammatory response in SA-ALI.

Current research highlights that cytokine activation and reactive oxygen formation, triggered by inflammation, are key drivers of sepsis [38]. Excessive inflammatory stimulation can trigger the burst of ROS within the organism. Oxidative stress is primarily caused by either excessive ROS production or insufficient antioxidant defense, underscoring the essential contribution of ROS to the pathogenesis of sepsis [39]. Moderate levels of ROS play a beneficial role in pathogen elimination. However, overproduction of ROS, a hallmark of sepsis, may lead to lung damage, exacerbate inflammatory responses at the cellular level, and intensify oxidative stress [17]. Therefore, strategies aimed at modulating oxidative stress are essential for SA-ALI. Our study found that ROS levels were significantly elevated during SA-ALI, and this increase was suppressed by CB treatment. Concomitantly, CB treatment reduced the level of the oxidative damage marker MDA, and enhanced antioxidant capacity, as evidenced by restored SOD activity and increased GSH level.

Research increasingly demonstrates that LPS stimulation leads to acute mitochondrial dysfunction and metabolic imbalance in macrophages [40,41,42]. In this process, the antioxidant defense system is impaired, and the mitochondrial electron transport chain is disrupted, leading to increased ROS production. Septic patients exhibit cellular energy depletion and tissue repair dysfunction due to mitochondrial abnormalities, outer membrane damage, and lowered respiratory chain enzyme activity [43]. Our experimental results demonstrated that the cellular OCR was impaired during SA-ALI, including reductions in basal respiration and proton leak, indicators related to cellular oxygen consumption. Conversely, CB treatment effectively restored these parameters. In addition, CB ameliorated the alterations in mitochondrial ROS and mitochondrial membrane potential. CB has been shown to reduce mitochondrial dysfunction in RAW264.7 cells triggered by LPS stimulation, which contributes to alleviating SA-ALI.

As a critical transcription factor, Nrf2 orchestrates redox homeostasis through inducing the production of diverse antioxidant enzymes [44]. The Nrf2 pathway activation has been demonstrated to attenuate sepsis-associated inflammation, oxidative burden, and mitochondrial abnormalities, thereby promoting better clinical prognosis [45]. After being activated, Nrf2 enters the nuclear compartment and interacts with the antioxidant response element, thereby triggering the expression of downstream genes like HO-1. HO-1 and its enzymatic products have been demonstrated to counteract both oxidative stress and inflammatory response. Evidence suggests that the Nrf2/HO-1 signaling helps reduce inflammation and oxidative damage in conditions associated with oxidative stress [46,47]. Our findings demonstrated that CB promotes the nuclear translocation of Nrf2 and upregulates the expression of Nrf2 and its downstream antioxidant-related proteins. Furthermore, the experimental verification using Nrf2 inhibitors and agonists further verified that the anti-inflammatory and antioxidant effects of CB are dependent on the activation of the Nrf2 signaling pathway. Nevertheless, this study still has several limitations. Although we have demonstrated that the Nrf2/HO-1 signaling pathway serves as the pivotal mechanism underlying the anti-ALI effects of CB, it remains unclear how CB activates the Nrf2 signaling cascade and what its exact molecular target is.

In conclusion, the research highlights the anti-inflammatory, antioxidant, and mitochondrial function-restoring effects of CB in SA-ALI, orchestrated through the Nrf2/HO-1 signaling cascade. Therefore, we hope this research can provide an experimental basis for advancing therapeutic interventions in SA-ALI.

## 5. Conclusions

This research systematically explored the therapeutic efficacy of CB in SA-ALI and its underlying mechanisms. Our findings demonstrate that CB triggers induction of the Nrf2/HO-1 signaling axis, thereby alleviating oxidative damage, inflammatory dysregulation and mitochondrial dysfunction and ultimately ameliorating SA-ALI.

## Figures and Tables

**Table 1:** The primer sequences involved in this experiment.

Gene	Forward	Reverse
*IL-6*	TCTATACCACTTCACAAGTCGGA	GAATTGCCATTGCACAACTCTTT
*IL-1β*	CTGTGACTCATGGGATGATGATG	CGGAGCCTGTAGTGCAGTTG
*IL-18*	CAACTTTGGCCGACTTCACTG	TGGGGTTCACTGGCACTTT
*TNF-α*	CTGAACTTCGGGGTGATCGG	GGCTTGTCACTCGAATTTTGAGA
*β-actin*	GGCTGTATTCCCCTCCATCG	CCAGTTGGTAACAATGCCATGT

## Data Availability

The original contributions presented in this study are included in the article/Supplementary Material. Further inquiries can be directed to the corresponding authors.

## Supplementary Materials

The following supporting information can be downloaded at: https://www.mdpi.com/article/10.3390/biomedicines14030729/s1, Table S1. Materials. Table S2. Antibodies.

## Abbreviations

The following abbreviations are used in this manuscript:

ALI	Acute lung injury
ARDS	Acute respiratory distress syndrome
ARE	Antioxidant response element
BALF	Bronchoalveolar lavage fluid
BSA	Bovine serum albumin
CB	Cumambrin B
Dex	Dexamethasone
DAPI	4′,6-diamidino-2-phenylindole
DMEM	Dulbecco’s modified Eagle medium
DMSO	Dimethyl sulfoxide
ELISA	Enzyme-linked immunosorbent assay
GSH	Glutathione
H3	Histone H3
H&E	Hematoxylin and eosin
HO-1	Heme oxygenase-1
*IL-6*	Interleukin-6
*IL-1β*	Interleukin-1*β*
*IL-18*	Interleukin-18
LPS	Lipopolysaccharide
MAPK	Mitogen-activated protein kinase
MDA	Malondialdehyde
MTT	Thiazolyl blue tetrazolium bromide
NF-κB	Nuclear factor kappa B
NO	Nitric oxide
NQO1	NAD(P)H: quinone oxidoreductase 1
Nrf2	Nuclear factor erythroid 2-related factor 2
OCR	Oxygen consumption rate
PBS	Phosphate-buffered saline
PVDF	Polyvinylidene fluoride
ROS	Reactive oxygen species
RT-qPCR	Real-time quantitative polymerase chain reaction
SA-ALI	Sepsis-associated acute lung injury
SOD	Superoxide dismutase
tBHQ	Tertiary butylhydroquinone
*TNF-α*	Tumor necrosis factor-*α*
