# Supplementary material for: Cumambrin B Alleviates Sepsis-Associated Acute Lung Injury by Activating the Nrf2/HO-1 Pathway
Source: Biomedicines. 2026 Mar 23;14(3):729. doi: 10.3390/biomedicines14030729 (PMC13024685; doi:10.3390/biomedicines14030729)
Supplement: Supplementary file 1 [file biomedicines-14-00729-s001.zip › biomedicines-4174267-supplementary.pdf]

Cumambrin B alleviates sepsis-associated acute lung injury by activating the Nrf2/HO-1 pathway

Yuemei Que [1,+], Dandan Ruan [1,+], Minxia Xu [1], Ying Nie [1], Guozheng Huang [2,*], Huajun Zhao [1,3,*] and Yanzi Yang [1,*]

[1] School of Pharmaceutical Sciences, Zhejiang Chinese Medical University, Hangzhou 311402, China; qym0925@zcmu.edu.cn (Y.M.Q.); rdd0602@zcmu.edu.cn (D.D.R.); xmx192315@163.com (M.X.X.); nancy163wyyx@163.com (Y.N.)

[2] School of Chemistry and Chemical Engineering, Anhui University of Technology, Anhui 243002, China; guozheng.huang@ahut.edu.cn

[3] Academy of Chinese Medical Sciences, Zhejiang Chinese Medical University, Hangzhou 310053, China; zhj@zcmu.edu.cn

[*] Correspondence: guozheng.huang@ahut.edu.cn (G.Z.H.); zhj@zcmu.edu.cn (H.J.Z.); yangyanzi@zcmu.edu.cn (Y.Z.Y.)

[+] These authors contributed equally to this work.

Table of Contents

Supplementary tables
Table S1. Materials.
Table S2. Antibodies.

Table S1:Materials

Name	Catalog No.	Manufacturer
Lipopolysaccharides(LPS)	L2880	Sigma-Aldrich (St. Louis, MO, USA)
Dexamethasone(Dex)	A601187-0005	Sangon Biotech Co., Ltd. (Shanghai, China)
Bicinchoninic acid (BCA) protein assay kit	P0011	Beyotime Biotechnology (Shanghai, China)
Malondialdehyde (MDA) assay kit	A003-1-2	Nanjing Jiancheng Bioengineering Institute, Co., Ltd. (Nanjing, China)
Glutathione (GSH) assay kit	A006-2-1	Nanjing Jiancheng Bioengineering Institute, Co., Ltd. (Nanjing, China)
Superoxide dismutase (SOD) assay kit	A001-3-2	Nanjing Jiancheng Bioengineering Institute, Co., Ltd. (Nanjing, China)
Goat Anti-Mouse IgG	ab150113	Abcam, Cambridge (MA, USA)
Dulbecco's modified eagle medium (DMDE)	11995065	Thermo Fisher Scientific (Waltham, MA, USA)
Methylthiazolyldiphenyl (MTT)	S19063	Yuanye Bio-Technology Co., Ltd. (Shanghai, China)
Total RNA extraction kit	K156001	Thermo Fisher Scientific (Waltham, MA, USA)
Evo M-MLV RT Premix for RT-qPCR	AG11706	Accurate Biotechnology, Co., Ltd. (Changsha, China)
Mouse interleukin-1β (IL-1β) ELISA kit	MM-0040M2	Wuhan Enzyme-Linked Biotechnology Co., Ltd. Wuhan, China)

Hematoxylin and eosin (H&E) staining kit	C0105	Beyotime Biotechnology (Shanghai, China)
DMSO	V900090	Sigma-Aldrich (St. Louis, MO, USA)
Beyo3D ™ Reactive Oxygen Species assay kit (DCFH-DA)	S1105S	Beyotime Biotechnology (Shanghai, China)
JC-1	C2005	Beyotime Biotechnology (Shanghai, China)
Oxygen consumption rate (OCR) assay kit	023004	Beijing Huawei Zhongyi Technology Co., Ltd. (Beijing, China)
Fetal bovine serum (FBS)	10099141	Thermo Fisher Scientific (Waltham, MA, USA)
MitoSOX Red mitochondrial superoxide indicator	S0061S	Beyotime Biotechnology (Shanghai, China)
4% Paraformaldehyde fix solution	P0099	Beyotime Biotechnology (Shanghai, China)
Triton X-100	TB0198	Beijing Solarbio Science Technology Co., Ltd. (Beijing, China)
Bovine serum albumin (BSA)	A600332-0100	Sangon Biotech Co., Ltd. (Shanghai, China)
DAPI	D9542	Sigma-Aldrich (St. Louis, MO, USA)
Nuclear and cytoplasmic protein extraction kit	P0028	Beyotime Biotechnology (Shanghai, China)
Clarity ™ Western ECL Substrate	102032321	Bio-Reference Laboratories, Inc. (Elmwood Park, NJ, USA)

Table S2:Antibodies

Name	Catalog No.	Manufacturer	Dilution for WB
GAPDH monoclonal antibody	60004-1	Cell Signaling Technology (Danvers, MA, USA)	1:1000
Rabbit anti-Nrf2 polyclonal antibody	ab62352	Abcam, Cambridge (MA, USA)	1:1000
Anti-NQO1 antibody	ab80588	Abcam, Cambridge (MA, USA)	1:1000
HO-1 rabbit mAb	43966S	Cell Signaling Technology (Danvers, MA, USA)	1:1000
Anti-Histone H3 antibody	ab309551	Abcam, Cambridge (MA, USA)	1:1000
